# Kutane Fernmetastase eines Pleuramesothelioms

**DOI:** 10.1007/s00105-025-05575-0

**Published:** 2025-10-08

**Authors:** Antigona Aliu, Katja Hohaus, Ulrich Peter Wehry, Zain Deeb, Frank Oellig, Thilo Gambichler, Sven-Niklas Burmann, Alexander Kreuter

**Affiliations:** 1 Klinik für Dermatologie, Venerologie und Allergologie, Helios St. Elisabeth Krankenhaus Oberhausen, Universität Witten/Herdecke, Josefstr. 3, 46045 Oberhausen, Deutschland; 2 Pathologie Mülheim an der Ruhr, Mülheim an der Ruhr, Deutschland; 3 Klinik für Dermatologie, Klinikum Dortmund, Universität Witten-Herdecke, Fakultät für Gesundheit/Medizinische Fakultät, Dortmund, Deutschland; 4 Klinik für Dermatologie, Christliches Klinikum Unna, Unna, Deutschland

**Keywords:** Hautmetastase, Mesotheliom, Immunhistologie, Fehldiagnose Plattenepithelkarzinom, Pleura, Cutaneous metastasis, Mesothelioma, Immunohistology, Misdiagnosis squamous cell carcinoma, Pleura

## Abstract

Kutane Metastasen eines Pleuramesothelioms sind selten und stellen aufgrund ihrer histopathologischen Heterogenität eine diagnostische Herausforderung dar. Wir präsentieren einen Fall, bei dem initial fälschlicherweise ein kutanes Plattenepithelkarzinom diagnostiziert wurde. Die korrekte Diagnose einer kutanen Metastasierung eines vorbekannten Pleuramesothelioms konnte erst durch eine differenzierte immunhistochemische Analyse gesichert werden. Die präzise histologische Klassifikation ist von hoher klinischer Relevanz, da das therapeutische Vorgehen insbesondere vom histologischen Subtyp sowie dem ECOG-Performance-Status des Patienten abhängt. Eine frühzeitige und exakte Diagnosestellung sowie die darauf basierende adäquate Therapieeinleitung sind prognostisch von entscheidender Bedeutung.

## Abstract (en)

Cutaneous metastasis of pleural mesothelioma is a rare manifestation and poses a diagnostic challenge due to its histopathological heterogeneity. We present a clinical case in which cutaneous metastasis of a known pleural mesothelioma was initially misdiagnosed. The correct diagnosis of cutaneous metastasis of a pleural mesothelioma was only confirmed through detailed immunohistochemical analysis. Precise histological classification is of high clinical relevance, as the therapeutic approach particularly depends on the histological subtype and the patient’s Eastern Cooperative Oncology Group (ECOG) performance status. Early and accurate diagnosis, as well as timely initiation of appropriate therapy, are prognostically important.

## Anamnese

Ein 85-jähriger Patient stellte sich mit einem innerhalb weniger Monate rasch progredienten kutanen Tumor im Bereich des rechten Kieferwinkels in unserer dermatologischen Ambulanz vor. Eine extern durchgeführte Probebiopsie ergab histologisch ein akantholytisches, mäßig differenziertes Plattenepithelkarzinom mit einem R1-Resektionsstatus. Klinisch bestand eine R2-Situation.

Zwei Jahre zuvor wurde bei dem Patienten ein linksseitiges, nichtoperables Pleuramesotheliom festgestellt. Die klinische Stadieneinteilung gemäß TNM-Klassifikation lautete zu diesem Zeitpunkt cT4, cN0, cM0 Stadium IIIB nach IASLC/UICC 8. Der lokal fortgeschrittene Tumor zeigte eine Infiltration in alle ipsilateralen Pleuraflächen (parietal, viszeral, mediastinal, diaphragmatisch) mit Pleuraerguss sowie eine Ausdehnung in die laterale Thoraxwand mit konsekutiver Destruktion der linken 7. Rippe im lateralen Abschnitt.

Im Rahmen einer interdisziplinären Tumorkonferenz wurde aufgrund des kompromittierenden Allgemeinzustandes eine palliative Chemotherapie mit Cisplatin und Pemetrexed im 21-Tage-Zyklus initiiert. Im weiteren Verlauf zeigten bildgebende Verlaufskontrollen mittels CT-Thorax und serieller Pleura- sowie Abdomensonographien eine Krankheitsstabilität ohne Tumorprogress, insbesondere keine infradiaphragmale Metastasierung. Die Therapie wurde aufgrund einer fortschreitenden chronischen Niereninsuffizienz (Stadium 3b nach KDIGO) auf eine Pemetrexed-Monotherapie umgestellt, jedoch infolge einer transfusionspflichtigen Panzytopenie abgebrochen. Angesichts des stabilen Krankheitsverlaufs, der deutlichen Blutbildveränderungen und des reduzierten Allgemeinzustandes wurde gemeinsam mit dem Patienten eine Therapiepause vereinbart.

## Hautbefund

**Abb. 1:**
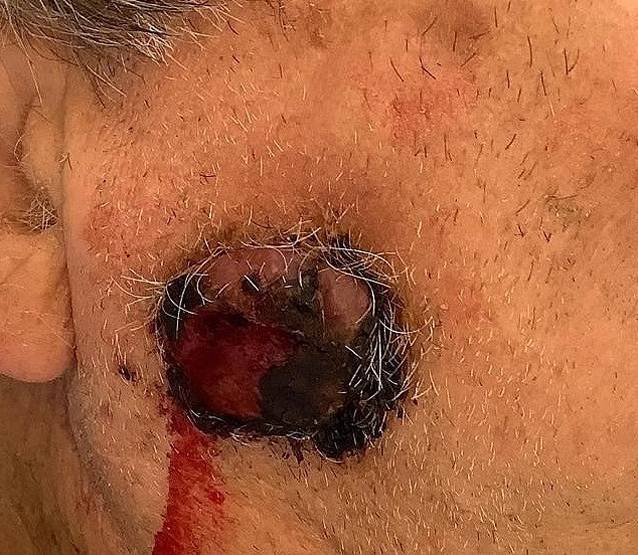
Seitliche klinische Aufnahme bei Erstvorstellung. Dargestellt ist ein etwa 25 mm großer, exophytisch wachsender, blutender Nodus im Bereich des rechten lateralen Unterkiefers auf Höhe des *Angulus mandibulae*.

Bei Erstvorstellung zeigte sich am rechten lateralen Unterkiefer im Bereich des *Angulus mandibulae*, auf Höhe des *M. masseter*, ein ca. 25 mm großer, gestielter, erythematöser, erosiver und blutender kutaner Nodus (Abb. 1). Die Läsion war nicht gegenüber dem Untergrund verschieblich und ohne Druckschmerz. Die regionalen Lymphknoten waren klinisch unauffällig.

## Diagnostik

**Abb. 2:**
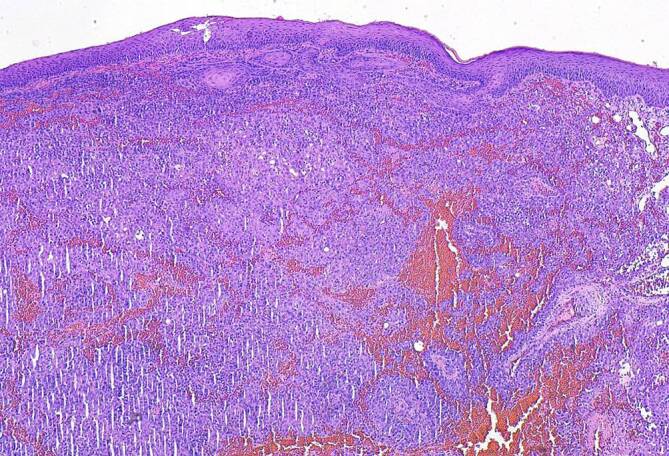
Hämatoxylin-Eosin-Färbung mit Darstellung eines mehrschichtig verhornten Plattenepithels der Haut. In der darunterliegenden Dermis zeigen sich ausgeprägte interstitielle Blutungen sowie eine Infiltration durch polymorphe, epitheloide, teils diskohäsiv wachsende Tumorzellen (Originalvergrößerung 100:1).

**Abb. 3:**
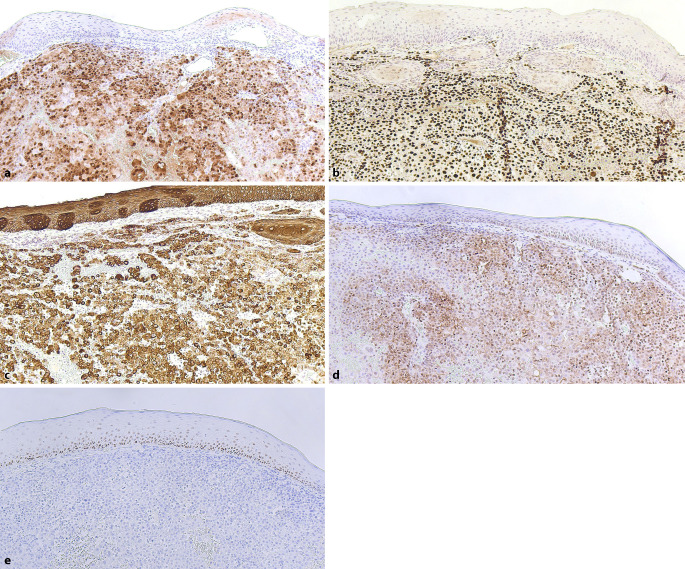
Immunhistochemische Untersuchung mit deutlicher Positivität für Calretinin (**a**); mit deutlicher, homogener nukleärer WT1-Expression (Wilms-Tumor-Protein) (**b**); mit Positivität für *CK5/6* (Cytokeratin 5/6) (**c**) sowie *D2-40* (Podoplanin) (**d**). Keine nachweisbare nukleäre oder zytoplasmatische p40-Expression in den Tumorzellen (**e**). Originalvergrößerung 100:1.

Histologisch zeigte sich nach durchgeführter histographisch kontrollierter Exzision ein exophytisch wachsender Tumor mit polymorphen, epitheloiden und teils diskohäsiv wachsenden Tumorzellen sowie ausgedehnten interstitiellen Hämorrhagien (Abb. 2). Die immunhistochemische Untersuchung ergab eine herdförmige Expression von Calretinin sowie eine homogene nukleäre Expression von WT1. Zudem waren CK5/6, D2-40 und AE1/AE3 positiv. Die Endothelien der Blutgefäße exprimierten CD31 und CD34. Die Tumorzellen zeigten keine Expression von S100, Melan A oder p40 (Abb. 3a–e).

## Diagnose

Die ergänzende Immunhistochemie führte zur Revision der initialen Diagnose und bestätigte eine kutane Metastase eines malignen Pleuramesothelioms (MPM). Nach der IASLC/UICC-Klassifikation (TNM, 8. Auflage, 2017) entsprach dies einem cT4 cN0 pM1-Befund im Stadium IVB.

## Therapie und Verlauf

Nach Sicherstellung eines R0-Resektionsstatus erfolgte die Defektdeckung mittels Dehnungsplastik in unserer dermatologischen Klinik. Der Fall wurde im Anschluss in der interdisziplinären Tumorkonferenz vorgestellt. Das Staging (CT von Hals bis Becken, cMRT) ergab keinen Anhalt für eine weitere Fernmetastasierung. Der Tumorkonferenzbeschluss beinhaltete die Reinitierung der Pemetrexed-Monotherapie sowie die Einbindung des Patienten in ein palliativ-supportives Behandlungskonzept.

## Diskussion

Das MPM ist ein seltener, hochmaligner Tumor, der typischerweise durch eine langjährige Asbestexposition insbesondere in Form von Blau- (Amphibol) oder Weißasbest (Chrysotil) in Deutschland verursacht wird [1].

Neben dem Pleuramesotheliom sind auch Mesotheliome anderer Lokalisationen wie Peritoneum, Perikard oder *Tunica vaginalis testis* bekannt, wobei die pleurale Form am häufigsten auftritt [2]. Gemäß der WHO-Klassifikation werden 3 histologische Subtypen unterschieden: epitheloide, biphasische und sarkomatoide Varianten, wobei letztere beide zu den nicht-epitheloiden Subtypen zusammengefasst werden [3, 4]. Diese Einteilung besitzt prognostische und therapeutische Relevanz [5]. In etwa 60 % der Fälle liegt ein epitheloider Subtyp vor [6].

Das MPM zeigt eine primär lokale Infiltration, kann aber über lymphogene und hämatogene Routen oder iatrogen durch direkte Ausbreitungswege (z. B. entlang von Biopsiekanälen oder Operationsnarben) auch Fernmetastasen bilden [6, 7]. Zu den häufigsten Metastasierungsorten zählen thorakale Lymphknoten, die kontralaterale Pleura, die Lunge und das Peritoneum [2]. Eine kutane Metastasierung im Rahmen eines MPM wie im vorliegenden Fall stellt eine seltene Manifestation dar. Bevorzugte Lokalisationen kutaner Metastasen sind typischerweise das Gesicht, die Kopfhaut sowie der Thorax [8]. Kutane Metastasen entwickeln sich meist wenige Monate nach Primärdiagnose, werden aber auch noch Jahre später beobachtet.

Collins et al. (2020) berichten über 2 Fälle solcher kutanen Manifestationen an der Stirn bei MPM sowie im Bereich der Oberlippe bei thorakalem und abdominellem malignem Mesotheliom. Abgesehen von den beiden 2020 publizierten Fällen kutaner Metastasen eines MPM in der Kopf-Hals-Region finden sich in der Literatur zwischen 1968 und 2010 lediglich 13 weitere dokumentierte Fälle – überwiegend bei männlichen Patienten (*n* = 11; 84,6 %) [9].

Besonders hervorzuheben ist das selten beschriebene Phänomen der isoradiotropen Reaktion – einer metastatischen Manifestation in zuvor bestrahlten Arealen. Dieses Muster wurde unter anderem bei einem 68-jährigen Patienten mit malignem Pleuramesotheliom und *V.-cava-superior*-Infiltration beobachtet, bei dem sich nach palliativer Bestrahlung eine kutane Metastase in der bestrahlten Brust- und Halsregion entwickelte [1]. Der pathophysiologische Mechanismus dieser Lokalisation ist bislang nicht abschließend geklärt. Diskutiert werden strahlungsinduzierte Veränderungen des lokalen Gewebemikromilieus, einschließlich einer gestörten Immunüberwachung, erhöhter vaskulärer Permeabilität sowie einer Modulation von Adhäsionsmolekülen, die das Tumorzellhoming begünstigen könnten [10]. Im Fall unseres Patienten lag keine entsprechende Vorbestrahlung des Primarius oder der betroffenen kutanen Region vor, sodass der Befund nicht im Sinne einer isoradiotropen Reaktion zu interpretieren war.

Die Diagnose eines epitheloiden Mesothelioms anhand einer Hautbiopsie ist aufgrund seiner histologischen Variabilität herausfordernd und muss von Differenzialdiagnosen wie Adenokarzinomen (Lunge, Kolon), Nierenzell- und Prostatakarzinomen sowie vaskulären Neoplasien wie Angiosarkomen abgegrenzt werden [11]. In unserem Fall erfolgte die extern gestellte Diagnose eines kutanen Plattenepithelkarzinoms ausschließlich auf Basis einer externen Probebiopsie ohne immunhistochemische Zusatzuntersuchungen. Insbesondere bei akantholytischen, mäßig differenzierten Tumoren gestaltet sich die feingewebliche Differenzierung ohne immunhistochemische Untersuchung häufig als besonders schwierig und kann leicht zu Fehldiagnosen führen. Die Tab. 1 zeigt differenzialdiagnostisch relevante Entitäten, bei denen die Immunhistochemie aufgrund histologischer Überschneidungen diagnostisch richtungsweisend ist.

**Tabelle 1:** Relevante klinische sowie histopathologische Differenzialdiagnosen mit Immunhistochemischen Charakteristika im Vergleich.

Entität	Positive IHC	Negative IHC
MPM	Calretinin WT1 D2-40 AE1/3 CK5/6 EMA	p40 CD34 CD31 BerEP4/MOC31
Plattenepithelkarzinom (SCC)	CK5/6 p40 EMA	Calretinin WT1 D2-40
Basalzellkarzinom (BCC)	AE1/3 BerEP4	EMA Calretinin
Angiosarkom	D2-40 CD34/CD31 ERG FLI1 WT1	AE1/3 Calretinin CK5/6
Ekkrines Karzinom	AE1/3 EMA BerEP4	D2-40 Calretinin
Karzinom-Metastasen	AE1/3 MOC31	D2-40 Calretinin

*IHC* Immunhistochemie, *MPM* Kutane Metastase eines epitheloiden Pleuramesothelioms

Für die Diagnose eines MPM empfiehlt die Internationale Mesotheliom Interest Group (IMIG) den Einsatz eines immunhistochemischen Panels mit mindestens 2 mesothelialen (z. B. Calretinin, Wilms-Tumor-Protein 1 [WT1], Podoplanin [D2-40], Cytokeratin 5/6 [CK5/6]) und 2 karzinomspezifischen Markern (z. B. CEA, TTF‑1, EpCAM, MOC-31). Für die Abgrenzung gegenüber Adenokarzinomen werden typischerweise folgende Marker eingesetzt: TTF‑1 („thyroid transcription factor-1“), welches hochspezifisch für pulmonale Adenokarzinome ist, CEA („carcinoembryonale antigen“) sowie MOC-31 („epithelial cell adhesion molecule“[EpCAM]-Antikörper) [2, 12, 13]. Bei Verdacht auf ein sarkomatoides Mesotheliom ist die Expression von breitbandigen Zytokeratinen (z. B. AE1/AE3) entscheidend, da sarkomatoide Tumoren mesothelialer Herkunft Zytokeratine exprimieren im Gegensatz zu vielen echten Sarkomen.

Bei nichtresezierbarem MPM mit epitheloider Histologie sowie bei nicht-epitheloiden Subtypen ohne hohen Remissionsdruck wird zunehmend die Kombination der Checkpointinhibitoren Nivolumab und Ipilimumab als Erstlinientherapie eingesetzt [2]. In der Zulassungsstudie *CheckMate743* zeigte sich für diese Immuntherapie ein Überlebensvorteil im Vergleich zur platinbasierten Chemotherapie. Zudem war die Symptomkontrolle (z. B. Fatigue, Dyspnoe, Schmerzen) verbessert und die Lebensqualität im Immuntherapiearm höher [1, 14]. Daher wird die Kombination aus Nivolumab und Ipilimumab bei beiden histologischen Subtypen als potenzielle Therapieoption betrachtet.

Bei resezierbarem epitheloidem MPM sowie bei nichtresezierbaren Tumoren mit hohem Remissionsdruck stellt die platinbasierte Chemotherapie mit Cisplatin und Pemetrexed weiterhin den empfohlenen Therapiestandard dar – wie auch im vorliegenden Fall [15, 16]. Als Zweitlinientherapie kann in solchen Fällen, bei nichtoperabler Krankheitssituation und nicht erfolgter Immuntherapie in der Erstlinie die Kombination aus Nivolumab und Ipilimumab im *Off-label-Use* erwogen werden. Bei Kontraindikationen gegenüber Cisplatin und/oder Pemetrexed kann Pemetrexed durch Vinorelbin oder Gemcitabin und Cisplatin ggf. durch Carboplatin ersetzt werden [17, 18].

## Fazit für die Praxis

- Bei neu aufgetretenen Hautveränderungen bei Patienten mit Pleuramesotheliom sollten auch kutane Metastasen als Differenzialdiagnose in Betracht gezogen werden, da ihre korrekte Identifikation entscheidend ist und den Behandlungsverlauf maßgeblich beeinflussen kann.
- Für die sichere immunhistochemische Abgrenzung eines Pleuramesothelioms von anderen malignen Entitäten, insbesondere pulmonalen und gastrointestinalen Adenokarzinomen, empfiehlt die Internationale Mesotheliom Interest Group (IMIG) die Anwendung eines Markerpanels mit mindestens 2 mesothelialen und 2 karzinomspezifischen Markern, um eine diagnostische Präzision und Sicherheit zu gewährleisten.
- Die Immuntherapie mit Kombinationen aus Ipilimumab und Nivolumab etabliert sich zunehmend als therapeutische Option bei MPM und zeigt insbesondere in fortgeschrittenen und nichtresezierbaren Fällen ein vielversprechendes klinisches Potenzial.

## References

[1] Abou Shahla W, Khoury DM, Saade D (2024) Cutaneous metastasis from pleural mesothelioma at site of radiation therapy: Case report and review of the literature. JAAD Case Rep 54:37–40 39583059 10.1016/j.jdcr.2024.08.033 PMC11585786

[2] Metzenmacher M, Aigner C, Curioni-Fontecedro A et al (2023) Malignes Pleuramesotheliom (ICD-10: C45.0) – Leitlinie zur Diagnostik und Therapie hämatologischer und onkologischer Erkrankungen. Deutsche Gesellschaft für Hämatologie und Medizinische Onkologie (DGHO). https://www.onkopedia.com/de/onkopedia/guidelines/pleuramesotheliom/@@guideline/html/index.html#litID0E3KAG. Zugegriffen: 2015

[3] Galateau-Salle F, Churg A, Roggli V et al (2016) World Health Organization Committee for Tumors of the Pleura. The 2015 World Health Organization Classification of Tumors of the Pleura: Advances since the 2004 Classification. J Thorac Oncol 11(2):142–154 26811225 10.1016/j.jtho.2015.11.005

[4] Beasley MB, Galateau-Salle F, Dacic S (2021) Pleural mesothelioma classification update. Virchows Arch 478:59–72 33475835 10.1007/s00428-021-03031-7

[5] Dacic S (2022) Pleural mesothelioma classification—update and challenges. Mod Pathol 35(Suppl 1):51–56 34465883 10.1038/s41379-021-00895-7

[6] Ward RE, Ali SA, Kuhar M (2017) Epithelioid malignant mesothelioma metastatic to the skin: a case report and review of the literature. J Cutan Pathol 44(12):1057–1063 28800180 10.1111/cup.13026

[7] Mori T, Yamamoto T (2021) Skin metastasis of malignant mesothelioma. An Bras Dermatol 96(5):642–643 34304935 10.1016/j.abd.2020.07.020 PMC8441512

[8] Elbahaie AM, Kamel DE, Lawrence J et al (2009) Late cutaneous metastases to the face from malignant pleural mesothelioma: a case report and review of the literature. World J Surg Oncol 7:84 19900274 10.1186/1477-7819-7-84 PMC2777157

[9] Collins K, Nagarajan P, Aung PP (2020) Distant cutaneous metastasis of malignant epithelioid mesothelioma. J Cutan Pathol 48(7):902–907 33258154 10.1111/cup.13927

[10] Ruocco E, Caccavale S, Siano M et al (2014) Radiation port cutaneous metastases: a further example of immunocompromised district. Indian J Dermatol 59(3):302–303 24891670 10.4103/0019-5154.131420 PMC4037960

[11] Abban C, Viglione M (2009) Peritoneal mesothelioma presenting as a skin nodule. J Cutan 36(6):675–679 10.1111/j.1600-0560.2008.01094.x 19515047

[12] Terada T (2011) Skin metastasis of pleural epithelioid malignant mesothelioma. Appl Immunohistochem Mol Morphol 19(1):92–93 20861794 10.1097/PAI.0b013e3181e94121

[13] Sinn K, Mosleh B, Hoda MA (2021) Malignant pleural mesothelioma: recent developments. Curr Opin Oncol 33(1):80–86 33186182 10.1097/CCO.0000000000000697

[14] Baas P, Scherpereel A, Nowak AK et al (2021) First-line nivolumab plus ipilimumab in unresectable malignant pleural mesothelioma (CheckMate 743): a multicentre, randomised, open-label, phase 3 trial. Lancet 397:375–386 33485464 10.1016/S0140-6736(20)32714-8

[15] Vogelzang NJ, Rusthoven JJ, Symanowski J et al (2003) Phase III study of pemetrexed in combination with cisplatin versus cisplatin alone in patients with malignant pleural mesothelioma. J Clin Oncol 21:2636–2644 12860938 10.1200/JCO.2003.11.136

[16] Ceresoli GL, Aerts JG, Dziadziuszko R et al (2019) Tumour Treating Fields in combination with pemetrexed and cisplatin or carboplatin as first-line treatment for unresectable malignant pleural mesothelioma (STELLAR): a multicentre, single-arm phase 2 trial. Lancet Oncol 20(12):1702–1709 31628016 10.1016/S1470-2045(19)30532-7

[17] de Gooijer CJ, van der Noort V, Stigt JA et al (2021) Switch-maintenance gemcitabine after first-line chemotherapy in patients with malignant mesothelioma (NVALT19): an investigator-initiated, randomised, open-label, phase 2 trial. Lancet Respir Med 9(6):585–592 33515500 10.1016/S2213-2600(20)30362-3

[18] Zauderer MG, Kass SL, Woo K et al (2014) Vinorelbine and gemcitabine as second- or third-line therapy for malignant pleural mesothelioma. Lung Cancer 84(3):271–274 24690410 10.1016/j.lungcan.2014.03.006 PMC4343315

[19] Osama MA, Gaur K, Chatterjee P et al (2023) Acantholytic Squamous Cell Carcinoma: a Diagnostic Pitfall on Cytology. Indian J Surg Oncol 14(4):963–967 38187856 10.1007/s13193-023-01811-y PMC10767014

[20] Mardi K, Singh N (2014) Acantholytic squamous cell carcinoma of the oral cavity: A rare entity. J Oral Maxillofac Pathol 18(Suppl 1):S128–30 25364162 10.4103/0973-029X.141359 PMC4211221

[21] Yorita K, Tsuji K, Takano Y et al (2018) Acantholytic squamous cell carcinoma of the lung with marked lymphogenous metastases and high titers of myeloperoxidase-antineutrophil cytoplasmic antibodies: a case report. Bmc Cancer 18:300 29548309 10.1186/s12885-018-4218-8 PMC5857100

[22] Beer TW, Shepherd P, Theaker JM (2000) Ber EP4 and epithelial membrane antigen aid distinction of basal cell, squamous cell and basosquamous carcinomas of the skin. Histopathology 37(3):218–223 10971697 10.1046/j.1365-2559.2000.00999.x

[23] Pisacane AM, Picciotto F, Risio M (2007) CD31 and CD34 expression as immunohistochemical markers of endothelial transdifferentiation in human cutaneous melanoma. Cell Oncol 29(1):59–66 17429142 10.1155/2007/486579 PMC4618198

[24] Dennis JL, Hvidsten TR, Wit EC et al (2005) Markers of adenocarcinoma characteristic of the site of origin: development of a diagnostic algorithm. Clin Cancer Res 11:3766–3772 15897574 10.1158/1078-0432.CCR-04-2236

[25] Bishop JA, Teruya-Feldstein J, Westra WH et al (2012) p40 (∆Np63) is superior to p63 for the diagnosis of pulmonary squamous cell carcinoma. Mod Pathol 25(3):405–415 22056955 10.1038/modpathol.2011.173

[26] Ordóñez NG (2005) D2-40 and podoplanin are highly specific and sensitive immunohistochemical markers of epithelioid malignant mesothelioma. Hum Pathol 36(4):372–380 15891998 10.1016/j.humpath.2005.01.019

[27] Fukunaga M (2005) Expression of D2-40 in lymphatic endothelium of normal tissues and in vascular tumours. Histopathology 46(4):396–402 15810951 10.1111/j.1365-2559.2005.02098.x

